# Sociobiology meets oncology: unraveling altruistic cooperation in cancer cells and its implications

**DOI:** 10.1038/s12276-024-01387-9

**Published:** 2025-01-07

**Authors:** Muhammad Sufyan bin Masroni, Evelyn Siew-Chuan Koay, Victor Kwan Min Lee, Siok Bian Ng, Soo Yong Tan, Karen Meiling Tan, Marco Archetti, Sai Mun Leong

**Affiliations:** 1 Department of Pathology, Yong Loo Lin School of Medicine, National University of Singapore, Singapore, Singapore; 2 NUS Centre for Cancer Research (N2CR), National University of Singapore, Singapore, Singapore; 3 Department of Pathology, National University Hospital, Singapore, Singapore; 4 Institute of Molecular and Cell Biology, Agency for Science, Technology and Research, Singapore, Singapore; 5 Singapore Institute for Clinical Sciences, Brenner Centre for Molecular Medicine, Singapore, Singapore; 6 Department of Laboratory Medicine, National University Hospital, Singapore, Singapore; 7 Department of Biology, Pennsylvania State University, University Park, PA USA

**Keywords:** Cancer, Cancer

## Abstract

Altruism, an act of benefiting others at a cost to the self, challenges our understanding of evolution. This Perspective delves into the importance of altruism in cancer cells and its implications for therapy. Against the backdrop of existing knowledge on various social organisms found in nature, we explore the mechanisms underlying the manifestation of altruism within breast tumors, revealing a complex interplay of seemingly counteracting cancer signaling pathways and processes that orchestrate the delicate balance between cost and benefit underlying altruistic cooperation. We also discuss how evolutionary game theory, coupled with contemporary molecular tools, may shed light on understudied mechanisms governing the dynamics of altruistic cooperation in cancer cells. Finally, we discuss how molecular insights gleaned from these mechanistic dissections may fuel advancements in our comprehension of altruism among cancer cells, with implications across multiple disciplines, offering innovative prospects for therapeutic strategies, molecular discoveries, and evolutionary investigations.

## Altruism in Cancer Cells Reveals New Therapeutic Insights

Altruism, or selfless behavior, has puzzled scientists for years, especially in the context of evolution. Traditionally, cancer cells are seen as selfish, growing uncontrollably. However, recent research suggests some cancer cells might act altruistically. They found that certain breast cancer cells produce substances that help neighboring cells survive chemotherapy, even though this slows their own growth. The study used breast cancer cell lines to observe these interactions. Researchers identified a subpopulation of cells with high levels of a molecule called miR-125b. These cells secrete proteins that protect other cells but grow more slowly themselves. This behavior fits the definition of biological altruism, where one organism incurs a cost to benefit others. The findings suggest that understanding these altruistic behaviors in cancer could lead to new treatment strategies. This summary was initially drafted using artificial intelligence, then revised and fact-checked by the author.

## Introduction: altruism across life and cancer

Altruism, the selfless act of benefiting others at the actor’s expense^1^, has intrigued scientists and philosophers throughout history, posing challenges to our understanding of evolution. Charles Darwin himself grappled with its paradoxical existence^2^. Early works by Petr Kropotkin, Warder Allee and J.B.S. Haldane set the stage^3^, but biologists such as W.D. Hamilton^4,5^ and Robert Trivers^6^ in the 1960s and 1970s provided groundbreaking insights into the evolutionary roots of altruism. By studying altruism, researchers continue to decipher the mysteries of social behavior and cooperation in biological systems.

Altruism has two interpretations: everyday altruism, characterized by conscious selfless acts without expecting anything in return^7^, and biological altruism, a behavior that is costly to the actor and beneficial to the recipient, with the cost and benefit defined on the basis of the lifetime direct fitness consequences of a behavior^8^. Our review focuses on biological altruism and explores its mechanisms and evolutionary implications, rather than extensively covering everyday altruism.

**Fig. 1:**
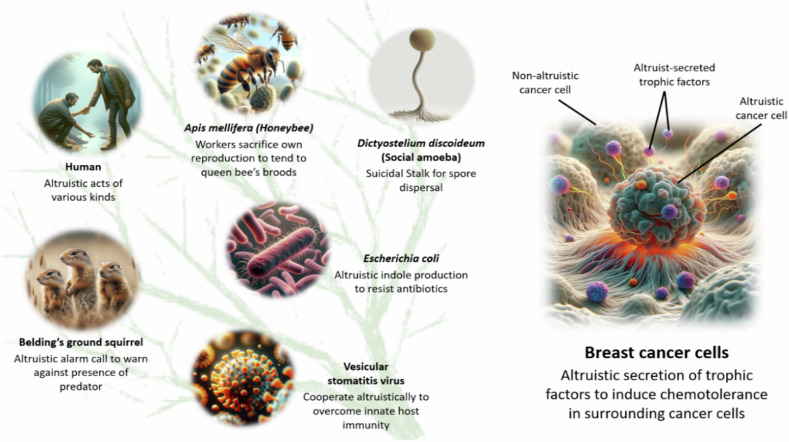
Altruism across diverse life forms and types of cancer. Nature is replete with examples of altruistic cooperation across the tree of life, from viruses to unicellular prokaryotes (*Escherichia coli*) and eukaryotes (social amoebae) and to multicellular eukaryotes (honeybees, Belding’s ground squirrels, humans). As part of multicellular organisms such as humans, cancer cells do not fit into the traditional tree of life, but they have recently been shown to manifest altruistic behavior through the secretion of trophic factors to induce increased tolerance of the surrounding cancer cells during exposure to the chemotherapeutic agent taxane^24^. (Parts of the figure were generated via ChatGPT 4.).

Remarkable examples of altruistic behavior can be observed across a wide range of organisms (Fig. 1). These include viruses such as vesicular stomatitis virus, which cooperate to overcome host cell immunity^9^; bacteria such as *Escherichia coli*, which sacrifice growth for antibiotic resistance^10^; and myxobacteria and social amoebae, which form suicidal stalks for the benefit of spore dispersal^11,12^. Altruism extends to higher organisms such as eusocial insects (bees, ants, termites) with cooperative activities like reproductive altruism^13^, and birds with cooperative breeding^14^. Even mammals such as Belding’s ground squirrel display altruistic behaviors by alerting group members to potential danger, despite risking their own safety^15^. In humans, altruism is evident in activities such as blood donation, organ transplantation, charitable giving, and volunteering.

The theory of natural selection, perceived by Darwin himself to be challenged by altruism^2^, has firmly established itself in evolutionary biology over the past 160 plus years. It has also been found to be relevant in cancer research, particularly in understanding clonal evolution during tumor growth and therapy resistance, owing to Peter Nowell’s seminal paper^16^. Despite altruism being a well-studied phenomenon in nature, its presence and exploration within oncology remain relatively uncharted.

Recent findings of possible altruistic behavior in cancer cells offer a unique avenue for investigation, bridging social behavior and cooperation with cancer biology. Historically, cancer has been framed primarily as a model of selfish behavior at the cellular level, where cells gain mutations that enable unchecked growth, evading the usual constraints of multicellular cooperation. While it has been acknowledged for some time that cancer cells cooperate in a mutualistic or synergistic manner^17–23^, emerging research suggests that cancer cells may not only act selfishly but also exhibit forms of cooperation akin to biological altruism, whereby some cancer cells incur fitness costs to benefit the overall tumor population^24^ (Fig. 1; Supplementary Box 1).

Many cancer cells may, for example, respond to environmental stressors such as chemotherapy by reducing their own proliferation and secreting factors that increase the survival and growth of neighboring cells, effectively sacrificing their own potential for the benefit of overall resistance. Another hypothetical example could involve certain cancer cells entering a state of senescence, halting their own growth but releasing factors that protect or sustain nearby cells. These behaviors align with the principles of biological altruism; altruistic cancer cells incur a fitness cost, yet their actions ultimately promote the survival and expansion of the tumor population. This approach is distinct from mutualistic cooperation, where all involved cells benefit equally. In altruistic behavior, the cost is borne by the altruistic cells, while the larger tumor population reaps the benefit.

In particular, our discovery that some cancer cells secrete prosurvival factors for the benefit of neighboring cells^24^ provides the impetus for studying altruism within tumors. In this context, altruism can be interpreted as a survival strategy that emerges under specific environmental pressures, such as the hostile conditions created by immune responses or chemotherapy, where the tumor acts as a quasi-social system. This form of cooperation may help the tumor maintain heterogeneity, a key factor in therapy resistance and disease progression.

Although cancer cells may not fit the traditional tree of life, they may provide a compelling model for studying altruism and cooperation, offering valuable insights into tumor evolution, therapeutic responses, and the broader understanding of cooperative dynamics in diverse life forms.

## Altruistic cooperation within a tumor

### Cancer: from multicellularity to unicellular mode of survival

The evolution of multicellularity, a significant milestone in Earth’s history, involves the transition from self-centered unicellular entities to cooperative groups^25^. This shift requires suppressing competition among selfish entities and aligning individual cell fitness with group fitness^26^. Cancer development is often seen as the reverse—a breakdown in multicellularity, where cancer cells lose cooperative behaviors and revert to unicellular survival^27,28^. Some people argue that cancer cells exhibit competitive growth, similar to that of unicellular organisms such as bacteria and yeast^29^. However, this overlooks the cooperative behaviors displayed by unicellular microorganisms, which engage in communication, collaboration, and various activities, including altruistic acts^30^. In some instances, the occurrence of fitness disadvantages to the cooperator was empirically demonstrated^10,31^, thus establishing the nature of the cooperative act as altruistic. These examples show that unicellular life balances competitive and cooperative functions, suggesting that cooperation may still play a role in the evolution and survival of cancer cells, even after they transition away from multicellularity.

### Cancer cell cooperation: clues from intratumor heterogeneity

Since the 1980s, evidence has indicated that tumor cells cooperate within the tumor. Heppner and her team first highlighted this through the observation of phenotypic heterogeneity, which hinted at the existence of different tumor subclones. Coculture of these various subclones revealed that different combinations of subclones could lead to enhanced or impeded tumor growth, suggesting the presence of cooperative or antagonistic interactions among tumor cells^32–36^. However, these early demonstrations of clonal cooperation lack mechanistic insights because of the limited availability of molecular tools^32–36^. Heterogenous clones within the tumors have been likened to functional castes in “superorganisms” such as bees and ants, whereby social cooperation between different castes underlies the success of these “superorganisms”^37^. The resurgence of interest in clonal cooperation in the early 2010s revealed how interactions between distinct subpopulations affect tumorigenesis, metastasis, and therapeutic outcomes^17–22^. Studies describe the exchange of beneficial factors between subpopulations (termed mutualism) or how different tumor clones cooperate to underlie new oncogenic phenotypes (termed synergism) to explain intratumor heterogeneity^23^. However, social aspects of tumors remain underexplored, missing the opportunity to uncover various cooperative interactions, including altruism, which is not driven by self-interest (unlike mutualism)^38^ and challenging natural selection.

Prior to the discovery of altruism in breast tumors^24^, possible hints of altruistic behavior among cancer cells existed. “Public goods cooperation” involves certain cancer cells secreting products that benefit neighboring cancer cells via angiogenesis, growth signals, and tissue invasion^28,39^. In a xenograft study, analysis of tumors with different compositions of a slower-growing IL-11-overexpressing subclone and a faster-growing LOXL3-overexpressing subclone demonstrated that the former may serve an altruistic function^20^. While LOXL3-overexpressing cells expanded rapidly, they did not drive tumor growth. Moreover, the IL-11-overexpressing subclone facilitated increased tumor growth in the presence of LOXL3, indicating that LOXL3-overexpressing cells benefited from the IL-11-overexpressing cells. Clonal analysis further revealed that LOXL3-overexpressing subclones outcompeted IL-11 in IL-11/LOXL3 tumors, indicating a fitness disadvantage for IL-11-overexpressing subclones^20^. These findings suggest that the IL-11-overexpressing subclone may act altruistically in a tumor with a fast-growing subclone free-riding on its ‘public goods’ contribution, thus incurring a fitness cost while supporting overall tumor growth. Similar results in glioblastoma models revealed minor subpopulations as drivers of tumor growth and heterogeneity^40^, suggesting the possibility of altruistic cooperation within tumors, although an in-depth analysis of fitness costs on the part of altruistic cells is lacking.

### Altruism among breast cancer cells: recent insights

We have described possible altruistic behavior in breast cancer cells, where a small subpopulation of cells increases tolerance to the chemotherapeutic agent taxane at the population level^24^. This study focused on a specific subpopulation of cancer cells characterized by high expression of the noncoding RNA miR-125b. These cells secrete proteins that activate PI3K, conferring survival advantages to neighboring cells when exposed to taxane. However, compared with their counterparts, miR-125b-high cancer cells experience growth retardation and cell cycle arrest, resulting in a fitness disadvantage. To elucidate the social interaction between these two subpopulations, we analyzed their relative proportions during and after chemotherapy exposure and compared them with those of a control group devoid of the miR-125b-high subpopulation. This analysis yielded two parameters, relative survival and relative fitness, which allowed us to assess the benefits and costs of this interaction^24^ (Supplementary Box 2).

Using a four-way classification matrix of social behavior adapted from Hamilton^4,5^ and West et al.^8^, we identified possible altruistic interactions in breast cancer cells both in vitro and in vivo^24^ (Supplementary Box 2). This finding is further supported by the observation of miR-125b-high and miR-125b-low cancer cell growth in isolation or coculture, a well-accepted method for detecting cooperation or cheating^41^ and protective communal interaction^10^ in microorganisms. Specifically, the minor subpopulation of miR-125b-high cancer cells provides a communal protective effect through altruistic behavior^24^, a phenomenon not previously detailed in other studies, hinting at altruistic cooperation in cancer. The observed interactions are consistent with behaviors that could thus be interpreted as altruistic, although alternative mechanisms, such as bet hedging (a strategy to reduce risk by diversifying responses to unpredictable environments)^42^ or group selection (evolutionary selection acting on groups rather than individuals)^8^, are also plausible explanations; this highlights the complexity of interpreting cooperative behavior in cancer cells.

## Mechanistic dissection of altruistic benefits and costs using cancer cells

### Altruistic benefits and costs: two faces, same body

In altruism, distinguishing between benefits to others and costs to the altruistic individual is crucial. Owing to self-incurred costs, altruistic cooperation differs from other social interactions, such as mutual benefits and commensalism (Supplementary Box 1). The study of social behaviors in microbes has significantly contributed to our understanding of the mechanisms underlying the emergence of altruistic benefits and costs (Table 1). Knowing these underlying mechanisms is pivotal for deriving a holistic understanding of the evolution of altruism, as it is through changes in these mechanisms that altruistic behavior can evolve and persist^43^. Microbes, with short generation times and genetic manipulability, provide a model for understanding the mechanisms underlying altruistic benefits and costs^30^.

**Table 1:** Altruistic fitness benefits and costs in social organisms and cancer cells.

Altruism observed in	Altruism mediated through	Fitness benefits (to recipients)	Fitness cost (to altruistic actors)	References
Vesicular stomatitis virus	Protein M	Blocking of interferon from infected cells produces a shared benefit to local viruses, allowing them to infect neighboring cells	Reduced replication of viral altruists	Domingo-Calap et al.^9^
		Mechanism: Matrix protein M, produced by altruistic virus, suppresses host gene expression, thus preventing interferon production	Mechanism: Attributed to production of active M protein by the altruistic virus	
*Escherichia coli*	Indole	Turning on of drug efflux pumps and oxidative-stress protective mechanisms by secreted indole	Growth retardation due to indole secretion	Lee et al.^10^
		Mechanism: Drug-resistance mutations in altruistic bacteria lead to increased indole production	Mechanism: Attributed to production and secretion of indole by the altruistic bacteria	
*Salmonella typhimurium*	TTSS-1	Expression of the Type III secretion system virulence factors (TTSS-1) that inflame the gut, killing all intestinal microflora and most of the *S. typhimurium* cells in the vicinity. Remaining *S. typhimurium* is then free to exploit the situation and more effectively establish themselves in the gut.	Suicide due to inflammation caused by secretion of TTSS-1 by altruistic *S. typhimurium*.	Ackermann et al.^31^
		Mechanism: The *ttss-*1 gene is expressed in some *S. typhimurium* cells but not in others. The molecular mechanism of bistable *ttss-1* expression is not fully understood.		
*Dictyostelium discoideum* (Social amoeba)	Fruiting body stalk	Enhanced dispersal of spores from fruiting bodies held higher by stalk formed by altruistic amoeba	Formation of a suicidal stalk by altruistic amoeba	Schaap^44^
		Mechanism: During the differentiation of the aggregated amoeba slug, increased secretion of cAMP in the posterior region leads to the differentiation of prespore cells. DIF-1 produced by prespore cells induces some posterior cells to become prestalk cells (pstB and pstO). c-di-GMP synthesized by diguanylate cyclase A in prestalk cells acts as the signal for stalk cell differentiation, activating protein kinase A and promoting stalk gene expression.		
*Apis mellifera* (Honeybee)	JH, Vg etc.	Various, such as nest defense, trophallaxis (food sharing), brood nursing	Various, such as self-sacrifice during defense, sterility (reproductive altruism)	Corona et al.^45^
		Mechanism: Interplay between insulin-signaling pathway, juvenile hormone (JH) and vitellogenin (Vg). JH and Vg regulate caste differentiation and division of labor in honeybees.		
Mammals (including human)	OXT, AVP etc.	Various	Various	Anacker and Beery^46^; Ebstein et al.^100^; Madden and Clutton-Brock^101^; Soares et al.^102^*;* Sanderson et al.^103^
		Mechanism: Oxytocin (OXT), vasopressin (AVP), dopamine, serotonin, and glucocorticoids (GCs) are key endocrine players in cooperative behavior in mammals.		
Breast cancer cells	miR-125b	Increased miR-125b expression in altruistic cancer cells leads to increased secretion of IGFBP2 and CCL28, which, in turn, induce chemotolerance in neighboring nonaltruistic cancer cells (oncogenic process).	Increased miR-125b expression in altruistic cancer cells leads to impediment in G1-S cell cycle progression to self, due to miR-125b-mediated inhibition of cell cycle regulators such as CDK2, cyclin-A1 and E2F3 (tumor-suppressive process).	Masroni et al.^24^
		Mechanism: Secretion of IGFBP2 and CCL28 due to miR-125b expression is mediated through p65-dependent NF-κB signaling.	Mechanism: miR-125b-mediated cell cycle impediment is mediated through an NF-κB-independent function of IKKβ.	

Table 1 reveals that the relationships between fitness benefits and costs are often intertwined, similar to two sides of the same coin. For example, altruistic *Escherichia coli* cells increase indole production, benefiting neighbors, but suffer growth retardation due to mutations affecting indole production^10^. In altruistic suicide, fitness benefits (efficient spore dispersal) coexist with costs (stalk amoeba suicide) in multicellular fruiting bodies, with well-defined signaling events^44^. Obtaining such mechanistic insights is challenging in social insects and mammals, which lack molecular manipulability and an experimental framework. Despite this challenge, factors such as juvenile hormone and oxytocin have been shown to mediate cooperative behavior in honeybees and vertebrates^45,46^. The signaling mechanisms in social microbes are likely to differ from those in multicellular eukaryotes, limiting the direct application of microbial findings to understand social behaviors in eusocial insects, social mammals, and even humans.

### Oncogenic and tumor-suppressive processes: a peculiar alliance in altruism

Like microbes, human cancer cells offer tractable experimental systems that facilitate the study of social cooperation. This complements other models of social behavior and allows for profound molecular insights. For example, using breast cancer cell lines, we conducted experiments and investigated the mechanisms of altruistic interactions in breast cancer. Recent breast cancer cell models revealed a unique characteristic of miR-125b-high altruistic cells, namely, elevated expression of both oncogenes and tumor suppressors, indicating that the simultaneous activation of conflicting cancer processes may be pivotal in miR-125b-driven altruism^24^.

While microRNAs can have competing effects due to their multiple mRNA targets^47^, oncogenic and tumor-suppressive processes do not necessarily compete with each other; instead, they can jointly contribute to complex traits such as altruism. Specifically, miR-125b supports two crucial aspects of cancer cell altruism through differential NF-κB signaling: providing communal fitness benefits (oncogenic) and imposing fitness disadvantages on the cells themselves (tumor suppressors)^24^ (Table 1). This provides a sociobiological context for understanding the simultaneous activation of these opposing processes and reveals how the co-option of different parts of the same signaling pathway (NF-κB) achieves this phenomenon. Coexpression of oncogenes and tumor suppressors may also serve as a unique signature for identifying altruistic subpopulations in various cancers via single-cell RNA sequencing data. However, whether such paradoxical coexpression supports other complex cellular traits beyond altruism remains to be determined.

Cancer cells, like microbes, may thus offer valuable insights into the manifestation of altruism, shedding light on the intricate interplay of signaling events governing fitness benefits and costs within cellular systems. Using cancer cells as models provides convenience, accessibility, ethical advantages, and access to omics databases for studying social behavior. However, whether cancer cells are more relevant than unicellular microbes for understanding social behavior and its mechanisms in complex multicellular eukaryotes remains an open question. Importantly, cancer cell models cannot replicate the physiological, neural, or developmental mechanisms underlying altruistic behaviors in higher social eukaryotes.

## Dynamics of altruistic cooperation in cancer cells

The fitness cost of altruism challenges the evolution and persistence of altruism within populations. Earlier studies of cooperation focused on theoretical frameworks such as kin selection^48^ (helping relatives increase shared genetic survival), reciprocal cooperation^6^ (helping others with the expectation of receiving help in return), and other models, but the mechanisms governing the dynamics of altruistic cooperation remain unclear. Recently, researchers have used game theory and molecular experiments to study the mechanism regulating the dynamics of cooperation.

### Game theory

Interactions between organisms can be understood as ‘games’ where outcomes depend on their strategies and how they engage with others. Game theory, a mathematical tool, helps biologists study how these interactions influence evolution^49^. In evolutionary game theory, ‘payoffs’ refer to the benefits an individual gains, which affect the success of their strategies^50,51^. By using a ‘payoff matrix’, we can predict which strategies will lead to stable outcomes. Over time, natural selection favors strategies that are beneficial and likely to persist. Interestingly, altruistic behaviors, though seemingly at odds with the idea of survival of the fittest, can also become stable strategies^50^.

Experiments using games designed with reward systems have been carried out in rats, with mixed results, demonstrating either no cooperation or mutual cooperation^52–55^. Game theory has been applied to microorganisms, such as viruses^56^, yeast^57^, and bacteria^58,59^, under various conditions and in various environments. In cancer research, game theory has helped scientists understand how cancer cells cooperate by sharing resources such as metabolites^60,61^, oxygen^61^, hormones^62^, and growth factors^63–66^. One of the first experiments using game theory in cancer research involved insulin-like growth factor II (IGF2). Both cooperative and noncooperative cancer cells can exist together, as long as the effect of the growth factor does not increase in a simple, straight line in relation to the number of cooperative cells initially present (nonlinearity)^65^. This nonlinear effect matches predictions from models about how shared resources (public goods) work in evolutionary game theory^67–69^.

Additionally, game theory may offer insights into the regulatory mechanisms governing cooperation dynamics. In the breast cancer model mentioned earlier, spatial modeling revealed that cooperation breaks down when the benefits of helping others level off (concave effects) and when altruism is caused by genetic mutations^24^. To keep cooperation stable in such populations, cancer cells need to switch quickly between being altruistic and nonaltruistic, similar to how people adjust their behavior on the basis of others’ actions, known as ‘best response’ dynamics^70^, which are common in human interactions^71^. In breast cancer, this could occur through an epigenetic mechanism—changes in gene activity without altering the DNA—that allows cells to quickly change types. This led to the discovery that an epigenetic process involving P300/CBP-associated factor (PCAF) and Krüppel-like Factor 2 (KLF2) controls this switch^24^. Using game theory in experiments can provide valuable insights into how cooperative behavior works in social groups such as cancer cells.

### Mechanistic studies

To comprehend the dynamics of altruistic cooperation, two primary types of changes can be examined (Table 2):

- The first type involves a shift in an organism’s identity from defector to altruist or vice versa. This change may arise from mutations in one or more genes that directly or indirectly confer communal benefits on nonmutants in the population. For example, in *E. coli*, mutations in specific genes lead to increased indole production at the expense of the producer, fostering population-wide drug resistance and resulting in altruistic behavior^10^ (Table 2). Conversely, M protein mutations in VSV create cheaters that avoid the cost of inducing inflammation, preventing altruistic suicide due to inflammation^9^. Furthermore, epigenetic factors can influence social roles, as observed in honeybees, where feeding undetermined larvae royal jelly alters DNA methylation and acetylation patterns, causing larvae to develop into queens instead of reproductively altruistic workers^72,73^.
- The second type involves altering the proportion of altruists and defectors within a social population, often owing to factors secreted by one population to influence the social status of the other. For example, honeybee queens release pheromones that inhibit ovary development in altruistic workers, maintaining their reproductive sterility^74^. In social amoebae, pre-spores secrete the differentiation-inducing factor DIF-1, discouraging altruistic pre-stalk cells from becoming spores, thus preserving the 80:20% spore-to-stalk ratio in the fruiting body^75^.

**Table 2:** Mechanisms regulating the dynamics of altruistic cooperation in various social organisms and cancer cells.

Altruism observed in	Change in dynamics observed	Mechanism	References
Vesicular stomatitis virus (VSV)	Conversion from altruist to defector	Mutations in methionine 51 of the VSV matrix protein M, resulting in inactivation of this protein to suppress host gene expression and prevent interferon production. Δ51 mutant viruses are defectors, while those with wild-type M protein are altruists.	Domingo-Calap et al.^9^
*Escherichia coli*	Conversion from defector to altruist	Drug-resistance mutations lead to increased indole production. The increased indole then turns on the drug efflux pumps and oxidative-stress protective mechanisms in the recipients. Bacteria with these drug-resistant mutations are altruists.	Lee et al.^10^
*Salmonella typhimurium*	Regulation of percentage of altruists vs. defectors in the population	The regulator *hilA* controls the fraction of TTSS-1 positive altruists and TTSS-1 negative defectors. Mutations in *hilA* or *hilD* result in a population with purely TTSS-1 negative bacteria.	Sturm et al.^97^
*Dictyostelium discoideum* (Social amoeba)	Regulation of percentage of altruists vs. defectors in the population	The prespores secrete differentiation-inducing factor-1 (DIF-1) to prevent the altruistic prestalk cells from developing into spores. This mechanism helps to maintain an 80:20 spore-to-stalk cell ratio in the fruiting body formed	Williams^75^
*Apis mellifera* (Honeybee)	Conversion to defector or altruist	Queen or worker fate can be differentially altered by nutritional input. Silencing the expression of DNA methyltransferase Dnmt3 in newly hatched larvae led to development into queens rather than reproductive worker altruists.	Kucharski et al.^73^
*Apis mellifera* (honeybee)	Regulation of percentage of altruists vs. defectors in the population	Through the secretion of primer pheromones, such as CHCs (cuticular hydrocarbons) and other glandular compounds, the queen bee inhibits worker ovarian development, effectively preserving the workers as reproductive altruists.	Ge et al.^74^
*Heterocephalus glaber* (Naked mole-rat)	Conversion from defector to altruist	Increased estradiol in the queen’s feces after birth is disseminated to subordinates through coprophagy, which stimulated altruistic subordinates’ responses to pup vocalizations and increased cooperativity toward brood nursing.	Watarai et al.^98^
Breast cancer cells	Conversion from defector to altruist	miR-125b-high altruistic cells can be regenerated from the nonaltruistic fate via a PCAF/KLF2-mediated epigenetic mechanism	Masroni et al.^24^
	Regulation of percentage of altruists vs. defectors in the population	Diffusible products by altruistic cells (IGFBP2 and CCL28) activate a GAB1-PI3K-AKT-miR-125b-mediated “secrete-and-sense” circuit in neighboring miR-125b-low cells, preventing them from becoming altruists. This process constitutes a lateral inhibition mechanism, which maintains a sparse yet stable spatial organization of two social fates (miR-125b-high altruists and miR-125b-low defectors) within the cancer cell population	

In breast cancer, mechanisms akin to those seen in social organisms govern both types of changes in altruistic cooperation dynamics^24^. For the first type of change, miR-125b-high altruistic cells can transition from a nonaltruistic state through an epigenetic process mediated by KLF2 and PCAF. Interestingly, the insect counterpart of KLF2 (Kr-h1) has a role in caste-specific behavior in the ant species *Harpegnathos saltator*^76^, suggesting a role for Krüppel-like transcription factors in regulating the plasticity of social behavior.

For the second type of change, miR-125b-high altruistic cancer cells can secrete higher levels of diffusible proteins (IGFBP2 and CCL28) that not only confer benefits but also discourage neighboring cells from turning into altruistic cells. This phenomenon constitutes a lateral inhibition mechanism that maintains a sparse distribution of altruists within the cancer cell population^24^. Like honeybees and social amoebas^74,75^, diffusible factors from one subpopulation regulate the social fate of another. It remains to be proven whether other social organisms may use a similar negative feedback circuit between subpopulations, as observed in breast cancer cells.

Molecular advancements and sequencing technology have played crucial roles in understanding these mechanisms. For example, whole-genome sequencing technology is essential for discovering mutations leading to increased indole secretion in *E. coli*^10^. RNA interference has enabled the investigation of epigenetic regulators in eusocial insect models, while CRISPR/Cas9 holds promise for further insights into honeybee caste transitions^77,78^.

## Experimental tools for studying social interactions among cancer cells

Advanced sequencing techniques such as single-cell RNA sequencing provide insights into cell types, cell states, and cell‒cell interactions at single-cell resolution, surpassing traditional bulk sequencing methods. Recent innovations in spatial transcriptomics^79,80^, which map gene activity within the physical location of cells in tissue, add spatial context to single-cell RNA sequencing. This allows researchers to examine individual cells within their specific tissue environments, especially in patient samples. This spatial information enhances our understanding of tissue architecture (e.g., solid vs. hematopoietic liquid cancers) and molecular organization, revealing proximity, potential interactions, and the expression of ligand‒receptor pairs among cell types.

The spatial arrangement of cells is important for studying how they interact within tissues. In tissues where some cells may act altruistically, these cells may be few in number because helping others comes at a fitness cost. These altruistic cells may be spread out and release signals (ligands) that affect nearby nonaltruistic cells, which have matching receptors to respond to these ligands. Different types of interactions, such as cooperation or altruism, may have unique spatial patterns and signal‒receptor setups. Researchers can use this information to create ‘spatial fingerprints’ that can help identify and study these social behaviors by considering factors such as cell proportions, distances, and signal types to identify the different types of social interactions.

**Fig. 2:**
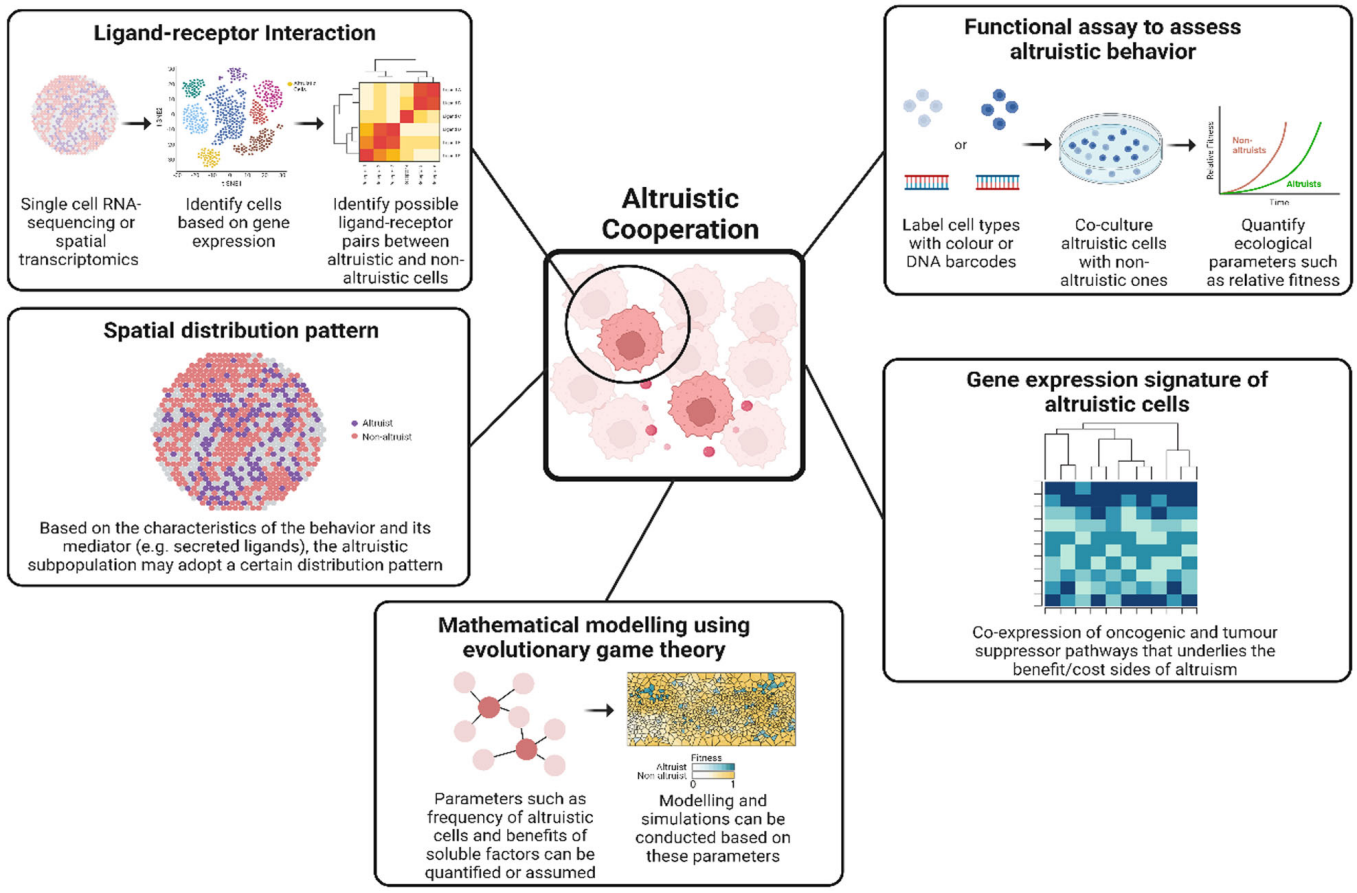
Experimental tools that can be used to study social interactions among cancer cells. Cutting edge technologies such as spatial profiling can be used in conjunction with single-cell omics profiling, ligand–receptor profiling, mathematical modeling via evolutionary game theory and function assays involving coculture to identify and characterize social interactions such as altruistic cooperation in tumor samples and cell lines of various cancer types. (Figure generated via Biorender).

Tools such as spatial transcriptomics provide a detailed, multilayer view of tissues, helping us understand their makeup and potential social interactions. First, by analyzing gene expression in individual cells, we can measure the diversity within a tumor via Shannon’s index^81^, which is a measure used in ecology to quantify the diversity of a community. Next, we can predict how these diverse cells interact with each other by looking at patterns such as their location and closeness. Spatial techniques also help explore these interactions and the signals (such as secreted molecules) that mediate them. When combined with RNA sequencing, this approach can identify genes linked to specific interaction patterns. It can also yield hypotheses about the molecular mechanisms underlying social behaviors, guide functional studies to test these ideas, or provide data for mathematical models on the basis of evolutionary game theory (Fig. 2).

## Cancer cell altruism: implications and future work

Reimagining cancer cells through sociobiology opens new opportunities across various fields, including oncology, molecular and cell biology, and evolutionary biology.

### Oncology

**Fig. 3:**
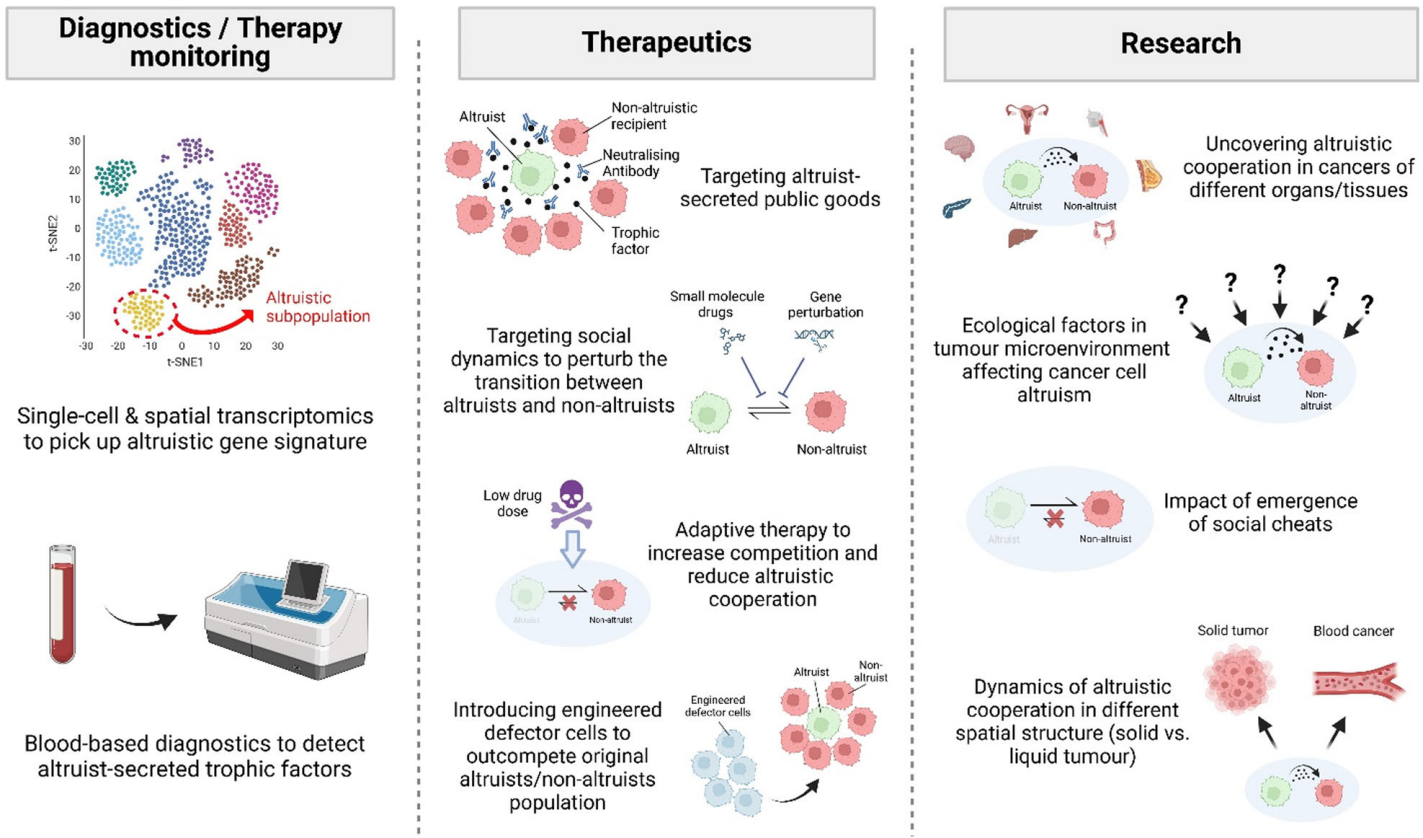
Implications of cancer cell altruism for the field of oncology. Implications of cancer cell altruism in oncology. This figure illustrates how understanding altruistic cooperation in cancer cells can lead to potential advancements in the development of innovative therapeutics and diagnostic techniques, alongside the exploration of new avenues in cancer research. (Figure generated via Biorender).
    The development of therapeutic strategies aimed at disrupting altruistic cooperation may also face challenges, as multiple mechanisms may be involved, and tumor cells may develop resistance by shifting to alternative cooperative behaviors. However, despite these challenges, deciphering the mechanisms underlying the social interactions between cancer cell subpopulations will increase the array of therapeutic options and potentially lead to new therapeutic developments.

Studying cooperation among microbes has led to important medical insights, such as the development of antibiotic resistance^10,82^, biofilm formation^83^, and cooperative virulence^84^. Understanding the nature and mechanism of social cooperation among cancer cells may likewise provide medical benefits in the following areas (Fig. 3):

- *Diagnostics & therapy monitoring*
  The observation that a relatively small subpopulation of miR-125b-high altruistic cells can engender population-wide effects^24^ offers a caveat to current diagnostic endeavors focused solely on genes or epigenetic candidates found in the majority of the tumor cell population. The detection of minor altruistic subpopulations may become more feasible with advancements in single-cell sequencing and spatial transcriptomics. As mentioned above, molecular signatures such as mixed oncogene/tumor-suppressor activation within a single cancer cell may indicate altruistic cooperation. The use of liquid biopsy-based diagnostics may help to circumvent issues associated with small subpopulation sizes within the tumor. For example, we can detect an increase in the blood concentration of altruist-associated diffusible factors or circulating tumor cells with altruism-associated expression signatures, as demonstrated in breast cancer^24^.
- *Therapeutics*

- *Targeting public goods-mediated fitness benefits* - Altruistic cancer cells secrete public goods such as IGFBP2 and CCL28, increasing chemotolerance in neighboring cells^24^. Impeding intercellular communication, e.g., neutralizing antibodies against these public goods, may constitute an avenue to sensitizing the tumor to chemotherapeutic agents. Therapies targeting diffusible factors such as IGFBP2 and CCL28 may be less susceptible to evolution of refractoriness than conventional cytotoxic therapies are, as the former allows for weaker selection pressure for resistance than the latter, which directly affects cell survival^39,85^.
- *Targeting social dynamics within the tumor* - Understanding the mechanism underlying the persistence of the altruistic cancer cells, despite the fitness disadvantage, may lead to insights into how we can modulate the altruist–defector composition for therapeutic gain. As demonstrated in breast cancer, hindering altruistic regeneration through epigenetic perturbation may thus be a feasible strategy to disrupt these altruist‒defector dynamics^24^, alter social composition and impede altruistic interactions before therapeutically attacking the entire tumor^23,39^.
  - *Adaptive therapy* - An application of game theory in cancer therapy involves altering drug dosages to encourage competition between cancer cell subtypes. This approach, known as ‘adaptive therapy’^86–88^, involves using lower drug doses to stimulate competition and reduce cooperation (including perhaps altruistic ones) among cancer subclones, potentially hindering resistance development. Another potential tactic involves manipulating a tumor’s selection pressure to favor the nonaltruistic/cheater subpopulation to thrive at the expense of the altruists^89^.
  - *Engineering defector cells* - An alternative involves modifying tumor cells by disabling genes responsible for vital growth factors (such as IGFBP2 and CCL28 in breast cancer^24^ or IGF2, IL6 and PDGFD^90^). These altered cells, when reintroduced into the original tumor, could have a competitive edge. They thrive by utilizing the growth factors produced by unmodified cells, outperforming the original subpopulation that can give rise to altruists via epigenetic mechanisms. Over time, this competition results in a tumor predominantly composed of these modified cells. When exposed to chemotherapy, these tumors likely collapse due to the absence of essential growth factors. This strategy leverages clonal selection to our benefit, leading to the spread of a subclone that does not produce growth factors, thus resulting in the breakdown of cooperation within the tumor — a “tragedy of the commons”^91^ (a situation where individuals, acting in their own self-interest, overuse and deplete a shared resource, leading to harm for the entire group) at the cellular level.

(c) *Research*

The establishment of a cancer cell altruistic model enables numerous avenues of investigation:

- Researchers can now explore altruistic interactions in different settings, including primary and metastatic sites; various cancer types; and their roles in tumor growth, metastasis, angiogenesis, and immune evasion.
- This model also facilitates the exploration of ecological factors in the tumor microenvironment (e.g., the nature of the extracellular matrix, proximity to blood vessels, and composition of immune cells) that either encourage or inhibit cancer cell altruism. Consequently, it enables a deeper understanding of the ecological elements influencing cancer progression and offers the possibility of identifying novel therapeutic targets.
- Another important aspect to explore is the impact of social cheats on altruistic cooperation. Studying how cancer cells may exploit altruism without contributing to it can provide valuable insights into the stability of altruistic behaviors within cancer cells.
- By examining the dynamics of altruistic cooperation in tumors with different spatial structures, such as solid vs. liquid (hematopoietic) tumors, we can gain a deeper understanding of tumor behavior and potentially identify strategies to manipulate or disrupt these cooperative processes.

### Molecular and cell biology

In contrast to fields such as developmental biology and immunology, our understanding of the molecular and cellular mechanisms in sociobiology remains relatively modest. Leveraging cancer cell models to investigate altruistic and other cooperative behaviors has the potential to make significant contributions to uncovering novel signaling phenomena that govern social interactions.

#### Understanding altruism signaling

Although the classification of cancer-regulatory genes as either “oncogenes” or “tumor-suppressor genes” has significantly contributed to our understanding of tumor biology, the discovery of genes that exhibit both functions has challenged conventional frameworks, often leading to overlooked or unpublished observations when gene behavior does not align with simplistic classifications^47,92,93^. These “double agent” genes may play dual roles in oncogenesis depending on specific cellular contexts, and multiple factors can determine whether a gene has a net oncogenic or tumor-suppressive effect^47^. We previously showed that the coactivation of these pathways in altruistic behavior is facilitated by miR-125b^24^. This finding represents a significant observation in which a single gene can simultaneously exhibit both oncogenic and tumor-suppressive functions within the same cancer cells without mutually negating each other. The fact that a signaling mechanism enables a single gene (miR-125b) to concurrently exhibit both oncogenic and tumor-suppressive functions within the same cancer cells^24^ is a novel and unprecedented observation. Further investigations into this unique model, as well as other instances of cancer cell altruism, are likely to provide valuable insights into the poorly understood signaling mechanisms that underpin the coactivation of oncogenic and tumor-suppressive events.

#### Understanding novel mechanisms regulating social dynamics

Unlike classical Notch-Delta signaling, which mediates lateral inhibition^94–96^, an alternative paracrine signaling mechanism underlying lateral inhibition exists between breast cancer cells. As exemplified earlier, miR-125b-high cancer cells were found to secrete the diffusible proteins IGFBP2 and CCL28, which discourages neighboring miR-125b-low recipient cancer cells^24^ from becoming altruistic themselves. This cell‒cell communication relies on the GAB1-PI3K-AKT-miR-125b signaling circuit, forming a lateral inhibition mechanism that sustains a sparse distribution of miR-125b-high altruistic cells within the broader cancer cell population^24^. Additionally, modeling experiments revealed that by regulating the diffusion coefficient of the secreted proteins, it becomes possible to control the balance between altruistic and nonaltruistic cells. This lateral inhibition mechanism also limits the altruistic fate to a small subpopulation of cancer cells and is intricately tied to social fate determination within these cells. These findings may prompt further inquiry into whether this diffusible form of lateral inhibition may also play a role in social fate determination in other social organisms and various biological processes.

#### Genetics vs. epigenetics in altruism

The basis of altruism can be attributed to both genetic and epigenetic mechanisms. Mutations have been found to play a significant role in the emergence of altruistic or cheating behaviors, particularly in unicellular microbes^9,10,97^. However, in multicellular eukaryotes and cancer cells, social fate determination appears to rely more strongly on epigenetic and biochemical mechanisms^73,74,98^. Nonetheless, the prevalence of epigenetic and biochemical regulation raises the question of whether genetics still plays a role in regulating social fate determination in multicellular models. Cancer cells, characterized by a high mutation rate, provide an opportunity to investigate the genetic underpinnings of social behaviors. By utilizing this model, we can examine whether specific hotspot mutations exist that may lead to increased secretion of public goods, even at the expense of the individual’s own fitness. It is possible that such mutations are maintained within the population due to frequency-dependent selection^99^. Additionally, these hotspot mutations may recur more frequently under stressful conditions, similar to what has been observed in the bacterium *E. coli*^10^.

### Evolutionary biology

The study of cancer cell altruism has important implications for evolutionary biology. By examining the altruistic behaviors exhibited by cancer cells, we can gain insights into fundamental questions regarding altruism across the tree of life.

#### Composition of social subpopulations across species

Studying cancer cell altruism sheds light on how the regulation of diffusible factors influences the proportion of altruists to defectors in different social populations, as observed in honeybees^74^, naked mole rats^98^, and breast cancer cells^24^. This prompts inquiry into whether similar mechanisms exist in more complex social mammals, such as birds, mammals, and humans, which employ sophisticated cognitive abilities and sensory cues in communication.

#### Novel evolutionary insights

Cancer cells face unique challenges, as they shift from cooperating as part of a multicellular organism to focusing on their own survival by activating oncogenes and disabling tumor suppressors. Studying how some cancer cells take the “risk” of reverting to cooperative, tumor-suppressing behaviors helps us understand the switch between multicellularity and cooperation. This investigation may shed light on how multicellular organisms and cooperative behaviors evolve. Additionally, since cancers are derived from different tissues with unique developmental paths, understanding how cells cooperate in different cancers can reveal how certain developmental programs may promote cooperation in cancer.

#### Evolution of altruistic cooperation

Cancer cell lines are useful tools for studying what drives the evolution of altruism and the rise of selfish behavior in cells. By manipulating these cell lines and using experimental evolution along with advanced sequencing techniques, researchers can track markers of cooperation and molecular changes. Additionally, exploring kin selection in rapidly mutating, genomically unstable cancer cell populations offers a fascinating way to define “kinship” among different tumor subclones that evolve independently but live together in the same tumors over time.

## Conclusion

The reimagining of cancer cells through a sociobiology lens holds immense potential for advancing our understanding of social behavior and its mechanisms in complex organisms. The cancer cell model offers a unique opportunity to elucidate the intricate dynamics of altruism within a controlled and manipulable system. The molecular insights gained have the power to transform our comprehension of altruism and its evolution, with wide-ranging implications across disciplines, offering new possibilities for therapeutic strategies, molecular discoveries, and evolutionary investigations. By revealing the complex dynamics of cooperation and competition in cancer cells by cutting edge technologies such as spatial profiling, we can pave the way for innovative approaches in cancer treatment and gain a deeper understanding of fundamental social processes.

## Supplementary information

Supplementary Box 1 & Box 2
