# Supplementary material for: Sociobiology meets oncology: unraveling altruistic cooperation in cancer cells and its implications
Source: Exp Mol Med. 2025 Jan 7;57(1):30–40. doi: 10.1038/s12276-024-01387-9 (PMC11799181; doi:10.1038/s12276-024-01387-9)
Supplement: Supplementary file 1 — Supplementary Box 1 & Box 2 [file 12276_2024_1387_MOESM1_ESM.pdf]

Supplementary Box 1: Altruism vs. other social cooperations

Cancer cells existing in close proximity are known to interact amongst themselves or with the microenvironment. Positive interactions in the form of social cooperations can underlie tumour growth and survival. Some of these social cooperations, which are widely studied in the field of evolutionary ecology, are as follows:

Altruism: Altruistic behaviour involves individuals engaging in selfless behaviours that benefit others, even at a personal cost[8]. Altruistic individuals typically experience reduced proliferative success or survival compared to those acting solely in their self-interest.

Mutualism: Mutualism is defined as cooperation between different species[8]. In cancer research, mutualism is a form of cooperation where two or more different subpopulations within the tumour cooperate and produce factors that will benefits all involved parties[23]. In these relationships, each subpopulation gains advantages that outweigh any potential costs, creating a win-win scenario for all.

Synergism: Synergism, or synergistic cooperation, occurs when individuals or species collaborate, resulting in novel characteristics in the whole system that are absent if either population is present alone, without having an effect on the individual population[23]. In such cooperative interactions, efficiency and productivity are significantly enhanced.

Commensalism: Commensalism is a form of cooperation where one population can benefit another without being affected itself[23]. In this type of relationship, one party gains advantages while the other remains unaffected.

The key distinction between altruism, mutualism, synergism, and commensalism lies in whether fitness is incurred to the actors involved in the behaviour. Altruism involves self-sacrificial acts that reduce the altruist's own reproductive success or survival. In contrast, mutualism, synergism, and commensalism do not necessarily entail such a fitness cost to the cooperating parties. In mutualism, all involved parties benefit from each other; in synergism, they collectively achieve more than they could individually; and in commensalism, one party benefits while the other remains unaffected. These non-altruistic interactions can enhance overall fitness or resource utilisation without necessarily imposing a direct cost on any participant, making them distinct from altruism where the altruist pays a price for the benefit of others.

Supplementary Box 2: Testing for altruistic cooperation in chemotherapy-exposed cancer cells

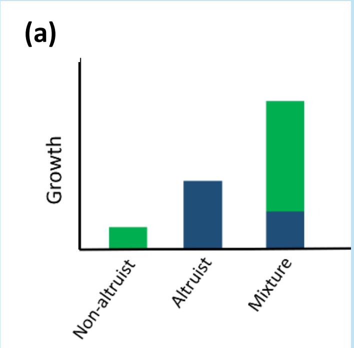

The gold standard test for cooperation[41] demonstrates the following: (i) that the non-altruists (or cheaters) benefit from the presence of the altruists when the two populations are mixed together; (ii) that the altruists, when grown separately from the non-altruists, show higher fitness than the latter; and (iii) that the altruists have lower fitness within the mixture as compared to when they are grown as a pure population (see Fig. a on the left).

However, this model becomes more complex when applied to the study of chemoresistance in breast tumours. Specifically, the fitness dynamics of altruists vs. non-altruists change depending on the presence or absence of the chemotherapeutic agent taxane[24]. In the absence of taxane, the non-altruists have higher fitness than the altruists. Conversely, under taxane exposure, the altruists display higher fitness than the non-altruists.

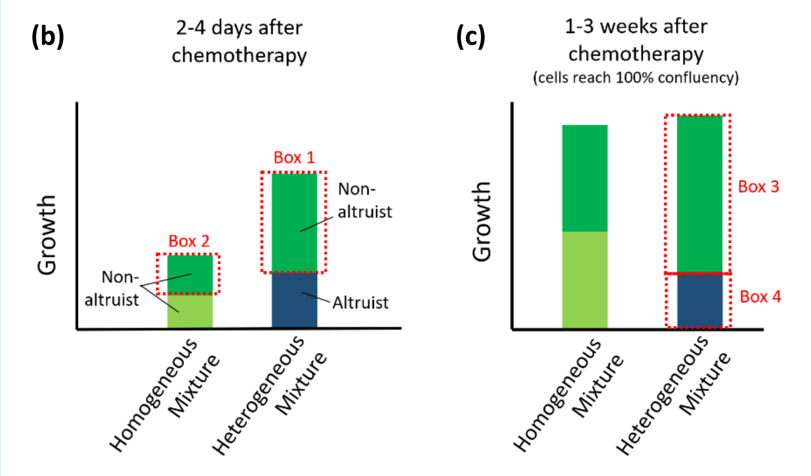

To adapt this model to the specific context of chemoresistance, we monitored the cell numbers of the altruists vs. non-altruists in different mixtures, via lineage tracing using fluorescence protein tagging, during and after taxane exposure (see Fig. b & c on the left).

We first examine the immediate effects of chemotherapy on the cells in the mixtures. Dividing the number of cells in Red Dotted Box 1 over that of Red Dotted Box 2 gives us the relative survival (RS). An RS of >1 means that the presence of the altruists benefitted the non-altruists within the heterogeneous mixture during exposure to chemotherapy, resulting in greater growth of the latter.

We next look at the fitness of each component of the heterogeneous mixture after allowing the surviving cells to regrow to full confluency. Dividing the number of cells in Red Dotted Box 3 over that of Red Dotted Box 4 gives us the relative fitness (RF). An RF of >1 means that the non-altruists outgrew the altruists within the heterogeneous mixture, indicating that the altruists suffered a fitness disadvantage post-treatment.

(d)

RF > 1<br>RS > 1<br>Altruism	RF = 1<br>RS < 1<br>Spite
RF = 1<br>RS > 1<br>Mutual Benefit	RF < 1<br>RS ≤ 1<br>Selfishness

Fitting the values of RS and RF into the four-way classification matrix of social behaviour (see Fig. d on the left) allows us to determine the nature of the social interaction between the two populations in the heterogeneous mixture. In this case, since both RS and RF are >1, the social interaction between the two populations is determined to be altruistic in nature.
